# The Use of Simulated Patients Is more Effective than Student Role Playing in Fostering Patient-Centred Attitudes during Communication Skills Training: A Mixed Method Study

**DOI:** 10.1155/2022/1498692

**Published:** 2022-12-17

**Authors:** Stanislaw Gorski, Anna Prokop-Dorner, Michal Pers, Agata Stalmach-Przygoda, Łukasz Malecki, Grzegorz Cebula, Katrien Bombeke

**Affiliations:** ^1^Department of Medical Education, Jagiellonian University Medical College, Krakow, Poland; ^2^Department of Medical Sociology, Jagiellonian University Medical College, Krakow, Poland; ^3^Department of Primary and Interdisciplinary Care, University of Antwerp, Antwerp, Belgium

## Abstract

**Objective:**

While simulated patients (SPs) are considered a standard tool in communication skills training, there is no evidence thus far of their comparative benefit to the more cost-effective option of student role playing. We compared the effectiveness of both approaches in developing patient-centred attitudes in students.

**Methods:**

We retrospectively compared students who participated in the clinical communication course (CCC), based on student role playing (CCCsp-, *n* = 160), to students who participated in the CCC with SPs (CCCsp+, *n* = 146), and students with no formal CCC (CCC-, *n* = 122). We used validated questionnaires to assess patient centredness. We also conducted focus group interviews (FGI) to better understand the impact of CCC with sp.

**Results:**

Students after the CCC with simulated patients achieved a significantly higher score in the patient-practitioner orientation scale than other groups (*p* < 0.001).

**Conclusions:**

There is a strong positive correlation between the implementation of simulated patients and patient-centred attitudes among students. Data from the FGI revealed that students perceived training with SP as more realistic, safe, and engaging than student role playing. *Practice Implications*. Our research provides evidence to justify costs and resources invested in simulated patient programs.

## 1. Introduction

Patient centredness (PCC) is at the core of modern concepts of professionalism in healthcare worldwide. In recent years, it has gained more prominence [1, 2] with growing policy and practice developments to promote patient-centred care through legislations and healthcare regulation. Most conceptual definitions of patient-centredness include health workers' communication skills as a core component. Likewise, recommendations regarding patient-centred learning goals and competencies in pre and postgraduate training tend to overlap with recommendations on teaching medical communication. Studies show that PCC is preferred by patients [3], among other things, improves trust in doctors [4], and improves positive lifestyle changes. It also reduces symptoms, the need for diagnostic tests, the number of hospitalisations and treatment costs [5, 6].

Research shows increased patient-centred attitude scores after the introduction of training communication skills (CST) [7–9]. These positive outcomes of CST are important given that attitudes are considerable determinants of behaviour according to the theory of planned behaviour (TPB) and the attitude-social influence-self-efficacy (ASE) model [10, 11]. According to ASE, human behaviour is driven by intentions influenced by external skills and barriers. Intentions are based on attitudes, self-efficacy, and social influence with strong attitudes being more predictive of behaviour than weak attitudes [11–13]. Teaching communication skills should therefore influence patient-centred behaviour by improving students' skills and shaping their attitudes.

For the successful development of communication skills, it is well established that medical educators should use experimental rather than purely didactic methods [14] to enable acquired skills to be integrated into clinical practice [15–17]. Simulation has become the basic educational strategy for the development of communication skills in medical education, despite some criticism [18]. Simulation allows learners develop skills and learn from their own mistakes in a safe environment in which they cannot harm the patient. It creates a learning environment in which adjustable levels of challenges can be achieved, exercises and tests are allowed, and feedback is optimised.

In teaching medical communication, commonly used simulation methods involve patients and role playing by students. Simulated patients (SPs) are persons trained to present a specific set of symptoms or roles. SP-based communication education allows learners to practice a wide variety of skills such as history taking, breaking bad news, dealing with aggression, and error disclosure. Frequently, trained simulated patients also provide feedback to learners. Simulated patients allow learners to practice skills on real people and receive feedback on their performance, while being encouraged to simultaneously reflect on their own practice. They are also suitable for both formative and summative assessments of communication skills [19, 20]. In most studies, the SP program is well perceived by students, but at the same time it is cost intensive and resource consuming.

Studies report a number of positive effects of training with SPs which include increased confidence of the students or healthcare workers [21, 22], improved communication performance as judged by the simulated patients [23, 24], and improved performance in standardised assessments [25]. On the other hand, Curtis et al. found that communication training of medical and nursing trainees with SP compared with usual education did not improve quality of communication about end-of-life care or quality of end-of-life care. Moreover, it was associated with a small increase in depressive symptoms in their palliative patients, which were attended after the training. This surprising phenomenon was partly explained by the biased selection of the trainees, not the training itself [26].

Another way to practice communication skills is role playing with fellow trainees via student role playing (RP). Trainees often complain that role playing with a person one knows feels artificial and thus express reluctance to participate. However, if RP training sessions are carefully designed and tutors are well trained, initial scepticism regarding participation in RP may be resolved [27]. On the other hand, this kind of training provides the additional benefit of trainees experiencing the role of the patient. It can help improve trainees' understanding of the complexity of the physician-patient interaction [28]. Student RP is also described as much more cost effective and easier to implement than SP role play [29].

Surprisingly, despite invested resources and significant differences in costs, most available research shows no clear benefit of SP compared to student role playing, beside students' satisfaction [30–32]. It is also important to note that despite repeating calls for investigations directly comparing these two educational methods [19, 33], the literature on this matter is scarce. Existing studies were mostly focused on observed behaviour in simulation circumstances (like OSCE exams), and examining perceived self-efficacy, but impact on attitudes has not been investigated so far. Therefore, we carried out a mixed-method study, using natural cohorts of medical students to compare the effectiveness of both methods of teaching clinical communication on patient-centred attitudes and to explore facilitators and barriers which occurred during the course.

## 2. Methods

### 2.1. The Clinical Communication Course Implementation

In 2014, a mandatory clinical communication course (CCC) was implemented into the curriculum of Jagiellonian University Medical College (JUMC) by the department of medical education, as a main part of laboratory training of clinical skills (LTCS) course. It had been implemented gradually, starting with student RP method for the first edition and then being replaced by SP role play in the next ones, which created a unique occasion for comparing the cohorts.

Prior to this, there were only elements of clinical communication presented in psychology and medical sociology classes. The course was designed based on the Calgary-Cambridge model [34, 35], and it was divided into 3 parts for 3 years of education (from the 3rd to 5th year of a 6-year medical program, consisting of 20 hours every year). The first part covered basic communication skills (communication skills during history taking and information sharing). During the second part of CCC, students encountered difficult communication issues such as patients' expectations, patients' aggression, sexual health issues, and breaking bad news. Students were practicing by performing role plays with each other. Finally, in their last part of CCC, use of clinical communication was implemented into high-fidelity patient simulation classes. In the next edition of the course, student role plays in the second and last part were replaced by practicing with simulated patients.

The aim of the study was to comparatively assess the effectiveness of the applied teaching tools in developing patient-centred attitude in students and to understand the factors determining the effectiveness of course delivery. In order to do so, quantitative (survey) and qualitative inquiry (focus group interview (FGI)) was sequentially conducted.

### 2.2. Quantitative Study

We retrospectively compared students who participated in the first edition of the CCC with student role playing without simulated patients involvement (CCCsp-, *n* = 160), participants of the second edition of the course who worked with SP (CCCsp+, *n* = 146), and students from the year before the implementation of CCC (CCC-, *n* = 122/202) in the case of patient-centred attitudes (PCA) and attitudes toward communication skills learning. We conducted a survey using an on-the-spot group survey technique in the final year of the medical program (before clinical clerkships). It was the 6th year in the CCC-group and 5th year in CCCsp- and CCCsp+ group. The reason for this difference was that, in 2016, the 6th year of studies was fully transformed into a clinical clerkship. In the CCCsp- and CCCsp+ groups, the preclinical part of the studies was compressed to 5 years, without decreasing the content significantly.

For comparison and measurement of students' attitudes, we used 3 validated questionnaires [36–39]. The patient-practitioner orientation scale (PPOS) and the Leeds attitude toward concordance II scale (LATCon II) were used to assess patient-centred attitudes. The communication skills attitude scale (CSAS) was used to assess attitudes toward learning communication skills. PPOS beside the total result is divided into two subscales—sharing (PPOS-S) and caring (PPOS-C). The students with higher scores in PPOS, PPOS-S and PPOS-C, and LATCon II presented better patient-centred attitudes. CSAS is divided into two subscales—positive attitudes (CSAS-P) and negative (CSAS-N). The higher scores in these subscales were connected with more positive or negative attitudes toward learning communication skills, respectively.

We obtained the authors' permissions to use and translate the scales into Polish. Forward translation was performed by an external company and expert panel, and back translation was performed by department of medical education's teachers and students from the university.

The basic characteristic of all scales can be found in Table 1.

Participation was voluntary, and students were fully informed about the study and that their refusal would not bear any consequence for them. Every student that participated in the study signed informed consent forms. Drop-off related to students' refusals was 25% in the CCCsp- group (52 students out of 212), 43% in the CCCsp+ group (110 students out of 256), and 39.6% in the CCC- group (80 students out of 202).

Statistical analysis was prepared in R v. 3.4.2 with the use of Chi-square, Mann–Whitney, and Kruskal-Wallis' tests with post hoc analysis by Dunn's test when necessary. Normality of distributions of the variables was evaluated using the Shapiro-Wilk test. A *p* value below 0.05 was considered statistically significant.

### 2.3. Qualitative Study

To complement the findings from the primary part of the study and to better understand the impact of CCC with simulated patients, we also conducted a focus group interview (FGI) [40] with students in their last year, who participated in the second edition of the course. One of the authors sent an invitation to students in their final semester informing them about a 60 to 90-minute-long interview on students' experiences in developing communication skills. The students were informed that the interview was to be conducted by an external researcher and remuneration for participation would be a voucher for a medical journal. Six students responded positively to the invitation and participated in the interview.

FGI was conducted according to the interview guide consisting of the main questions and discussion topics to be covered during the interview. The interview started with an individual task which aimed at qualitatively establishing participants' attitudes toward the patient-centred approach. Each participant was asked to reflect on their educational experiences and write down the three most crucial aspects of the patient-physician relationship. The individual work followed by a common discussion on what participants listed as the most important component of the relation.

Next, study participants were to map their most valuable experiences of acquiring communication skills on the timeline covering the whole study program and discuss the advantages and disadvantages of the teaching techniques applied at various stages of the learning process. FGI moderator probed the participants to share their individual educational experiences and discuss their opinions on strengths and weaknesses of the study program in regard to developing communication competences.

The interview was recorded and transcribed verbatim by an external transcriber. The data was inductively coded and code categorised. Through close analysis of the codes and the categories and the process of constant comparison of data, the themes were built [41]. The gathered material was rich, and the themes identified were saturated. The qualitative analysis was supported by MAXQDA 2018.

Finally, the results of both the quantitative and qualitative analysis were integrated, and common conclusions drawn.

### 2.4. Ethics and Funding

As suggested by the study design, the study team has positive patient-centred attitudes. The main researcher (SG) has been responsible for the coordination of the SP-program, besides his clinical work as a medical doctor. SG designed the study but was not involved in entering and statistical analysis of the data as these were done by external contractors and an expert statistician. The qualitative study (data collection and analysis) was conducted by a researcher from an external department (AP-D), not involved in the course and management of SPs in any way. To confront her interpretations of the data, during the process of data analysis, the qualitative findings were discussed with the research team. Transparent critical reflection in the continuous team discussions and external support by an independent colleague for the statistical analysis underlines the credibility of the quantitative findings.

The study was approved by the Bioethics Committee of the Jagiellonian University (nr 122.6120.321.2016). It was funded by Jagiellonian University Medical College, internal grant nr K/ZDS/007104.

## 3. Results

### 3.1. Quantitative Results

Apart from their age, groups were demographically homogenic. Students from the CCC-group were mostly 1 year older than in CCCsp- and CCCsp+ groups (23.49 v. 23.52 v. 24.82, *p* < 0.001), which is presented in Table 2. The reason for this difference was explained in the Method section.

**Figure 1:**
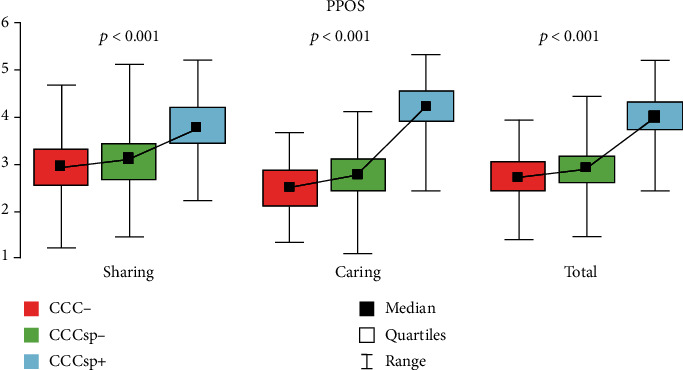
Comparison between groups in PPOS subscales.

CCCsp+ students reached a significantly higher score than CCCsp- and CCC-, respectively, in PPOS-S (3.8 v. 3.06 v. 2.95, *p* < 0.001) (Table 3, Figure 1). In PPOS-C, CCCsp+ scored higher than CCCsp-, who scored higher than CCC-, respectively, (4.2 v. 2.75 v. 2.52, *p* < 0.001). The same applies to PPOS (4 v. 2.91 v. 2.74, *p* < 0.001). In LATCon II, CSAS-N, and CSAS-P, we have not observed any significant differences. Results presented above are presented in Table 3.

### 3.2. Qualitative Results

The individuals who agreed to participate in the FGI (5 females and 1 male) represented various student groups. That means that, throughout the common study program, they have encountered slightly different educational stimuli resulting from i.e., working with different lecturers, mentors, and peers and seeing various patients. All FGI participants were highly engaged in the introductory task and shared their views openly and actively in the main part of the discussion. The discussion reflected both participants' individual observations and the collective experiences of their student groups.

When performing the individual task on the most crucial components of the patient-physician relationship and later discussing it in a group, FGI participants referred to notions conceptualised as patient centredness [42, 43]. They prioritised empathy, active listening, respect for patients, and providing them with a comfortable atmosphere. Students noted that a physician should be able to find time to talk to patients and explain their medical conditions and treatment options in a manner adjusted to their health competency. Students were convinced of the importance of focusing on patients' needs, giving them full attention and approaching everybody individually. One of the FGI participants explained that the most important thing for a physician is

“… to treat a patient as an individual, rather than another case (to whom you need to say: you need to do this, I recommend you to do that…), it helps building a trustful relationship talking exactly and only with this patient, with a very particular person, not with another case.” (Participant 6).

Students talked about their development of communication competencies as a dynamic and demanding process. Visualising the timeline of the 12 semester program, they mapped the milestones of the process. The following learning experiences were listed: role play with other students or simulated patients, observing medical professionals or peers approaching patients in a clinical context, conducting medical interviews with patients during group exercises, and learning about relatives or friends' experiences in medical settings. In order to understand which of those educational situations students found most beneficial and why, FG participants were encouraged to talk about the pluses and minuses of various educational approaches. All of the interviewed students declared having benefited largely from practicing with simulated patients. One of the students said,

“My group really liked those classes (with simulated patients). And for me, they were highly convincing. You could learn a lot, both practically and theoretically.” (Participant 4).

SP role playing was perceived by the study participants as one of the most advanced and informative forms of acquiring communication competencies that they encountered during the whole study process. Therefore, one of the FGI participants felt that practicing physician-patient communication skills in a framework of student pairs after patient simulation classes was a setback.

“And my group started to rebel. We had already practiced with the actors, we had already had experience with patients, and suddenly, we had to do role plays with other students again, and it was such a bad return.” (Participant 1).

#### 3.2.1. Students' Higher Motivation to Engage

The analysis of the material enabled us to identify three themes reflecting students' preference for practicing their communication skills with sp. The interviewed students talked about a higher level of motivation when participating in classes involving strangers and a higher level of engagement in the role-play situations when an actor impersonated a patient's situation in comparison to classes when role play was arranged between peers.

“In general, it (practicing with a simulated patient) was such a strong motivator. I wanted to make some effort and think more about what to say to the patient. You can also observe him/her more. Generally speaking, it was also a great form of classes.” (Participant 6).

“As for laboratory training of clinical skills (LTCS), for me that was what (facilitated the development of my communication skills) most. In the 4th year, as we had contact with actors from outside, we didn't have to do the role play with each other because it is hard to talk with a colleague from your group about some problems. Well, you immediately approach it differently, and the group gets distracted too because they don't approach it seriously.” (Participant 3).

In comparison to the group tasks of collecting a clinical interview with a patient, when practicing with a simulated patient, students felt far more responsible for the course of the conversation. One of the students confronted the level of responsibility perceived during those two training settings.

“It was a bit informative because it was also our first contact that we carried out from A to Z all the conversation alone. And not like in groups of five, when there was always someone to help out during the interview, and here, all responsibility was on each of us.” (Participant 2).

#### 3.2.2. Enhanced Realism of the Situation

FGI participants preferred practicing their communication skills with simulated patients rather than with their colleagues, also due to the former learning situations that seemed to be “more real”. Sociodemographic qualities of actors simulating patients were viewed as better reflecting the factual diversity of the patient population and their emotional expression as more adequate to real patient's reactions. One of the participants talked about what facilitated the positive effect of the trainings with SP.

“What made these actors different from our peers was that they were people of all ages. So, it was very real because there was some elderly lady and a guy younger than me, so you didn't have to imagine it at all. It was all really realistic.” (Participant1).

Moreover, the participants thought that actors were better trained to impersonate a patient than peers, who typically had only a few minutes to empathise with the patient's situation. In students' view, the fact that role of patients was played by an actor decreased the predictability of patient's reactions and therefore better reflected future encounters with patients.

“I think that everyone was not so much stressed but more motivated by the presence of some stranger and also that you did not know the reaction of that person, and it was not known how she would react to what we say or what we won't say and keep going and how it goes.” (Participant 2).

#### 3.2.3. Safety of the Training Context

The realism of SP role playing was also an opportunity to face some difficult behaviours of patients. One of the FGI participants said it was beneficial to face such a challenging situation with a patient in a training context initially.

“Among these situations with the actors, there were also difficult conversations, and if you worked with a good actor, and I just did, it was probably for me the most difficult conversation I have ever had because he reacted aggressively. Of course, not with a physical aggression, but verbal, which I have not encountered before and I think that when I would come across such a situation again, I would be better prepared. You should have such an experience, and you'd better experience it for the first time when practicing with an actor.” (Participant 1).

Another participant elaborated on the significance of a teacher's feedback and how it enhanced future contacts with real patients.

“Well, the doctor (teacher) was very engaged (in the training) and pointed out even the smallest mistake he noticed. And then, thanks to his feedback (during the training with simulated patients), when we went to the clinic and talked to patients, it was easier to see these errors and to correct what we had not noticed before.” (Participant 4).

## 4. Discussion

### 4.1. Discussion

Despite the evident demand, there are still relatively few articles studying the effectiveness of simulated patients compared to role playing in clinical communication courses for medical students. Our study has shown a significant increase in patient centredness measured by the PPOS questionnaire among medical students undergoing communication training with SPs in comparison to role playing by students themselves and students without communication skills training. To our knowledge, no other study has presented this kind of difference in outcomes of these training methods.

In the 2005 literature review by Lane and Rollnick, [33] only one article comparing SP and role play among medical students was found, and it did not show a significant between-group difference in communication skills during smoking cessation consultation [32] but showed greater student satisfaction with the course in the SP group. The authors of this review encouraged high-quality research in this area, which was repeated in the AMEE simulated patients guidelines [19].

Several studies were conducted at a single centre in Heidelberg, Germany and did not show significant differences in the groups who trained with SPs compared to role playing by students, excluding the subjective feeling of greater benefit of the course by students [44]. Moreover, one study showed superiority in expressing empathy in the role playing group on the OSCE exam compared to the group trained by SPs [45]. Cost-effectiveness studies conducted by Bosse et al. found significantly lower costs associated with role-playing groups when compared to SP groups without a significant difference in students' performance [29].

Another randomised study was performed in Australia among medical students, showing no significant differences in communication skills both in general assessment and in the assessment of separate components of consultations according to the Calgary-Cambridge checklist [46].

A recent observational comparative cohort study observed a small but statistically significant difference at the OSCE exam in favour of the group of medical students who received communication skills training with SP compared to the student role-playing group [47]. To our knowledge, this is the only article which shows any additional benefit apart from students' subjective feelings, in favour of the SP method among medical students. A very small study found superior outcomes for SP compared to role playing among 15 anaesthesiology residents with performance in simulated conditions assessed by faculty members. However, this population is not fully translatable to students' training [48].

Considering the scientific literature above, there is a significant discrepancy between scientific evidence and established educational practice [19]. The lack of evidence on the superiority of the training of clinical communication with simulated patients is noteworthy as the programs of simulated patients are described as very cost-intensive and organisationally demanding. Moreover, as mentioned before, there are reports about possible undesired outcomes of the training with SP for real patients, and there was no attempt to explain or replicate the study.

This enthusiasm despite adequate evidence can be partly explained by the subjective perception of students about the superiority of the SP training method compared to student role playing, described by several studies. We did not find data on the subjective feelings of academic teachers; it is an interesting research question to be addressed in the future, whether and to what extent it was their enthusiasm that influenced the spread of training with SP throughout the world.

Our study shows that this intuition may be right in a specific domain. The subjective feeling of students about the superiority of the use of SP might be related to their sense of change in attitudes and not only in a sense of increased skills. All cited studies above comparing simulated patients with role playing were based on OSCE results or behaviour assessment only. Our research fills this methodological gap, and our hypothesis is that the superiority of simulated patients is manifested in the improvement of attitudes and not necessarily in terms of behaviour presented in the examination conditions. Positive attitudes are crucial precursors of clinical performance. Moreover, none of the above-mentioned studies, directly comparing SPs and RP, applied a qualitative approach to better understand the process of acquiring communication skills among medical students. This uniqueness of research methods can partly explain the differences in the results compared to the existing literature [29, 44, 45].

Despite some limitations of the qualitative part of the study, including sample size, the data collected reflected various educational experiences from different student groups and provided us with important insights. In the course of designing and conducting the qualitative part, we paid attention to internal quality. To achieve authenticity and depth of the data, we asked at the beginning of FGI the participants to write down their individual reflections to prevent any group effects. Moreover, the moderator prompted participants' responses to receive detailed insights. The credibility of the data management and analysis has been taken care of by conducting the analytical procedures in a systematic manner and confronting the findings in the research team [49]. To support the findings, we illustrated them with quotations from the data. We identified three themes explaining students' preference of communication training with SP: motivation, realism, and safety. The interviewed students perceived SP method as more engaging than RP. This can be further linked to a much stronger perception of realism when training with SP compared to RP. It challenged students with unexpected situations but at the same time remained a safe training context allowing them to identify those aspects of communication competencies that require further improvements before encountering real patients.

Another limitation of the qualitative part might be the disproportion between female and male participants. A big body of research indicates a gender difference with regard to communication skills and preferences toward the patient-physician relationship [50]. In the quantitative part, though, there was no significant difference in gender among groups (Table 2).

The quantitative portion of the study is an observational cohort study with all the methodological limitations of this research method. Another weakness of the study is the lack of baseline (precourse) results for the questionnaires. In theory, the group which had the course with SP could have had a higher PPOS score from the beginning. On the other hand, the groups were relatively homogenous (Table 2), so we have no reason to suspect an important difference in precourse attitudes. As mentioned before, in the context of patient centredness, lack of significant differences in gender distribution is particularly important in this part of the study [50] .

The strength of the study is the relatively large number of surveyed students (428 in total) and a significant increase in the PPOS scale observed. This increase is much more significant in comparison with data comparing role-playing students to students who did not undergo an experimental learning course. This significance of the change increases the reliability of the results in the context of potential confounders.

Another limitation of this study, which applies virtually to all research related to patient centredness, is the lack of a universal definition for the term. This results in unclear measurement dimensions and heterogeneous usage of the term among different existing models [51]. We decided to measure patient-centred attitudes using a combination of 2 validated questionnaires available in the literature: Patient-practitioner orientation scale (PPOS) and Leeds attitude toward concordance II scale (LATCon II). These scales were chosen in order to cover the dimensions of patient centredness as defined by Mead and Bower [43]: ‘biopsychosocial perspective', ‘patient-as-person', ‘therapeutic alliance', and ‘sharing power and responsibility'. For the 5th dimension, i.e., ‘doctor-as-person', no scale has so far been developed for medical students. The communication skills attitude scale (CSAS) was used to measure students' attitudes toward communication skills learning. It was surprising to see no significant difference between groups, taking into account positive changes in attitude toward patient centredness. However, there are studies which show a decline in students' attitudes toward communication skills learning after a clinical communication course [52].

### 4.2. Conclusion

Communication skills training with simulated patients is more effective in inculcating patient centredness than role playing. The observed higher effectiveness of this method might stem from the more realistic, engaging, and safe learning context that it provides.

### 4.3. Practical Implications

Our research provides evidence to justify costs and resources invested in simulated patients programs.

## Figures and Tables

**Table 1:** Characteristics of questionnaires [36–39].

Questionnaire	Measured attitude	Number of items	Scoring	Score range	Example of item
LATCon II	Attitude toward concordance	20	4-point Likert scale: strongly agree to strongly disagree	0-36 (Sum)	Prescribing should take account of patients' expectations of treatment
PPOS-S	Attitude toward sharing information and decision-making process with the patient	9	6-point Likert scale: strongly disagree to strongly agree	1-6 (Mean)	The doctor is the one who should decide what gets talked about during a visit.
PPOS-C	Attitude toward enhancing the doctor-patient relationship and toward knowing the patient's psychosocial background	9		1-6 (Mean)	Although health care is less personal these days, this is a small price to pay for medical advances.
PPOS	Combination of PPOS-S and PPOS-C	18		1-6 (Mean)	
CSAS-P	Positive attitudes toward communication skills learning	13	5-point Likert scale: strongly disagree to strongly agree	13-65 (Sum)	In order to be a good doctor, I must have good communication skills
CSAS-N	Negative attitudes toward communication skills learning	13	5-point Likert scale: strongly disagree to strongly agree	13-65 (Sum)	I cannot see the point in learning communication skills

**Table 2:** Quantitative characteristics of the groups.

Parameter	Group			*p*
	CCC-	CCCsp+	CCCsp-	
Age (years)				
Mean ± SD	23.49 ± 1.08	23.52 ± 1.46	24.82 ± 0.85	*p* < 0.001^∗^VI > *V* + , *V*
Median	23	23	25	
Quartile	23-24	23-24	24-25	

Sex				
Male	62 (38.75%)	56 (38.36%)	31 (25.41%)	*p* = 0.036^∗^
Female	94 (58.75%)	85 (58.22%)	87 (71.31%)	
Not provided	4 (2.50%)	5 (3.42%)	4 (3.28%)	

**Table 3:** Comparison between groups in CSAS, LatCON II, and PPOS questionnaires.

Parameter	CCC- (VI)	CCCsp- (V)	CCCsp+ (V+)	*p* ^∗^
CSAS: positive scale				
Mean ± SD	46 ± 8.76	46.96 ± 8.4	45.88 ± 8.1	0.276
Median	47	48	46	NP
Quartiles	41-52	42-53	41-51	

CSAS: negative scale				
Mean ± SD	31.48 ± 5.9	31.22 ± 6.62	32.19 ± 5.2	0.141
Median	31	30.5	32	NP
Quartiles	27-35	27-35.25	29-35	

LatCON II				
Mean ± SD	40.47 ± 6.26	41.33 ± 5.04	42.56 ± 5.37	0.061
Median	41	42	42	NP
Quartiles	37-45.75	38-45	38-47	

PPOS: Sharing				
Mean ± SD	2.95 ± 0.62	3.06 ± 0.63	3.8 ± 0.58	<0.001
Median	2.94	3.11	3.78	*p*
Quartiles	2.56-3.33	2.67-3.44	3.44-4.22	*V* + >*V*, VI

PPOS: caring				
Mean ± SD	2.52 ± 0.48	2.75 ± 0.51	4.2 ± 0.51	<0.001
Median	2.5	2.78	4.22	NP
Quartiles	2.11-2.89	2.44-3.11	3.92-4.56	*V* + >*V* > VI

PPOS: total				
Mean ± SD	2.74 ± 0.47	2.91 ± 0.5	4 ± 0.44	<0.001
Median	2.72	2.92	4	*p*
Quartiles	2.44-3.06	2.61-3.18	3.72-4.33	*V* + >*V* > VI

## Data Availability

The data used to support the findings of this study are available from the corresponding author upon request (Excel sheets format).
